# Membrane trafficking in health and disease

**DOI:** 10.1242/dmm.043448

**Published:** 2020-04-30

**Authors:** Rebecca Yarwood, John Hellicar, Philip G. Woodman, Martin Lowe

**Affiliations:** School of Biological Sciences, Faculty of Biology, Medicine and Health, University of Manchester, Manchester, M13 9PT, UK

**Keywords:** Disease, Endocytic pathway, Genetic disorder, Membrane traffic, Secretory pathway, Vesicle

## Abstract

Membrane trafficking pathways are essential for the viability and growth of cells, and play a major role in the interaction of cells with their environment. In this At a Glance article and accompanying poster, we outline the major cellular trafficking pathways and discuss how defects in the function of the molecular machinery that mediates this transport lead to various diseases in humans. We also briefly discuss possible therapeutic approaches that may be used in the future treatment of trafficking-based disorders.

## Abstract (precis)

**Summary:** This At a Glance article and poster summarise the major intracellular membrane trafficking pathways and associated molecular machineries, and describe how defects in these give rise to disease in humans.

## Introduction

Membrane trafficking pathways are essential for cells to maintain critical functions, to grow, and to accommodate to their chemical and physical environment. Membrane flux through these pathways is high, and in specialised cells in some tissues can be enormous. For example, pancreatic acinar cells synthesise and secrete amylase, one of the many enzymes they produce, at a rate of approximately 0.5% of cellular protein mass per hour (Allfrey et al., 1953), while in Schwann cells, the rate of membrane protein export must correlate with the several thousand-fold expansion of the cell surface that occurs during myelination (Pereira et al., 2012). The population of cell surface proteins is constantly monitored and modified via the endocytic pathway. In some cells, endocytosis accounts for the complete turnover of surface membrane over a period of an hour or so (Steinman et al., 1976). Given such rates of trafficking, it is not surprising that even subtle alterations in transport caused by mutation or insufficiency of the trafficking machinery can impair cell function and lead to disease over the course of a lifetime.

**Figure:**
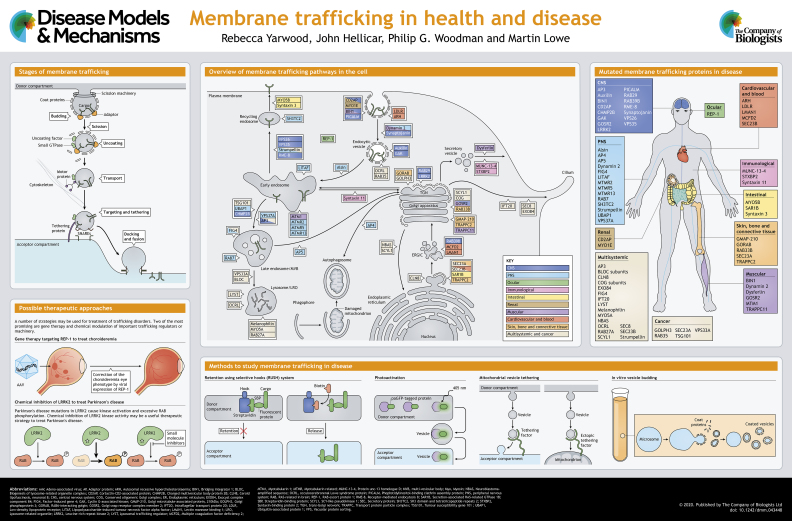

This At a Glance article describes the essential features of membrane trafficking pathways, including the crucial molecular events that drive transport. We identify instances where the mutation or loss of trafficking machinery components is associated with disease, and attempt to rationalise these effects. Several topics are not covered or are mentioned only briefly due to space limitations, including the folding and quality control of soluble or membrane-bound cargo, as exemplified by cystic fibrosis transmembrane conductance regulator (CFTR) in cystic fibrosis; motor proteins and their adaptors, which move vesicle-bound cargo around the cell; the biogenesis of mitochondria, peroxisomes, or non-membranous organelles; compartment-specific proteins that define essential organelle functions; non-vesicular lipid transport pathways; and exosome trafficking. Similarly, we only briefly discuss autophagy, which relies on membrane input from both the secretory and endocytic pathways and fusion of autophagosomes with lysosomes (Søreng et al., 2018).

## General principles of membrane trafficking

Transport of proteins between compartments is initiated by (1) selection of cargo and its segregation from resident proteins of the donor compartment by the action of ‘adaptors’; (2) encapsulation of cargo-bound adaptors within a protein scaffold or ‘coat’, which drives membrane deformation and ultimately scission to form a transport vesicle, or in some cases a tubular transport intermediate; (3) movement of the vesicle to the target compartment; (4) membrane tethering, in which the vesicle is drawn towards the target membrane by extended proteins/protein complexes that work in conjunction with RAB (RAS-related in brain) GTPases; and (5) docking and membrane fusion, in which the vesicle is first tightly attached to the target membrane, followed by merging of the lipid bilayers, both processes being mediated by soluble N-ethylmaleimide-sensitive-factor attachment protein receptor (SNARE) protein complexes and supported by accessory factors (see poster). While these steps are generic to transport reactions, the compartmental specificity of the components within protein families ensures transport fidelity. Much of our understanding of these processes stems from important experimental methodologies, which we describe in Box 1.

**Box 1. Experimental approaches used to study trafficking**

Many molecular cell biological approaches have been used to study membrane traffic. Historically, cell-free assays that reconstitute transport reactions (Balch et al., 1984) and yeast genetics (Novick and Schekman, 1979) provided great advances in identifying the crucial molecular components, and these approaches are still relevant today for dissecting transport mechanisms (see poster, ‘*In vitro* vesicle budding’). Cell culture models remain a powerful tool, with recent advances including growing cells in 3D to better mimic the tissue environment (Torras et al., 2018), the use of induced pluripotent stem cells that can be isolated from human patients and differentiated into any relevant cell type (Avior et al., 2016), and the use of stem cell-generated organoids, which provide a close approximation of tissue organisation in an *in vitro* setting (Lancaster and Huch, 2019; Rossi et al., 2018). Animal models also remain a valuable tool to study disease mechanisms attributable to trafficking defects, and have been used very successfully in this regard (see, for example, Smits et al., 2010). Analysis of human patients is also a powerful way to assess the functional relevance of gene products in a physiological setting, and provides a direct indication of the importance of trafficking factors for human health (FitzGerald et al., 2018).

A number of more recent or specialised approaches can be applied to the study of membrane traffic, some of which are highlighted in the poster. Various methods have been developed to allow synchronous transport along the secretory pathway (Kreis and Lodish, 1986; Chen et al., 2013; Kuismanen and Saraste, 1989; Rivera et al., 2000). One of the most commonly used is the retention using selective hooks (RUSH) system, in which synchronous transport is triggered by the addition of exogenous biotin, which triggers release of cargo from an organelle-resident ‘hook’ (Boncompain et al., 2012). The use of split-fluorescent protein technology allows researchers to assess delivery into secretory compartments (Feng et al., 2017). Here, cargo and organelle-resident proteins are separately tagged with two units of a fluorescent protein that, when combined, emit fluorescence, allowing for visualisation of cargo delivery to the organelle of interest. Photo-activation or photo-switching of fluorescently tagged cargo proteins or machinery can also be used to visualise transport dynamics (Sengupta and Lippincott-Schwartz, 2013).

Mitochondrial relocation is a useful tool for assessing protein-protein interactions, but more recently has been adapted to allow visualisation of vesicle tethering in intact cells. Here, tethering factors were artificially localised to mitochondria to allow direct visualisation of tethering by light and electron microscopy (Wong and Munro, 2014). Proximity biotinylation is a recently developed and widely used technology to identify closely associated proteins within cells. There are several variations of the method, which all rely on the promiscuous activity of a biotin ligase attached to any protein of interest, allowing for biotinylation of nearby proteins and their isolation and identification by mass spectrometry (Branon et al., 2018; Hung et al., 2016; Roux et al., 2012). The approach can be used in the context of membrane traffic to identify the machinery involved in particular trafficking reactions, cargo components of transport vesicles, or the protein complements of organelles within the endomembrane system. Quantitative proteomics can also be used to identify entire complements of secreted or plasma membrane proteins (Eichelbaum et al., 2012; Steinberg et al., 2013), allowing for unbiased and comprehensive analysis of how these protein complements may change in response to perturbation of various trafficking pathways.

## The secretory pathway

The biogenesis of most integral membrane proteins, secreted proteins and organelle content markers occurs at the endoplasmic reticulum (ER) (see poster). Correctly folded and post-translationally modified membrane-bound or lumenal cargoes are then selected for export by adaptor proteins that engage the coat protein complex (COP) II vesicle machinery, or by binding COPII directly (Jensen and Schekman, 2011; McCaughey and Stephens, 2018). COPII vesicle production is initiated when the ER-associated guanine nucleotide exchange factor (GEF) secretion protein (SEC) 12 (also known as PREB), activates secretion-associated RAS-related GTPase 1 (SAR1) and SAR1-GTP subsequently anchors to the membrane. The COPII coat is formed as SAR1 sequentially recruits multiple SEC23/SEC24 dimers, followed by SEC13/SEC31, to sequester adaptors and drive membrane deformation to produce COPII vesicles. These vesicles tether at and fuse with the ER-Golgi intermediate compartment (ERGIC), from which they are delivered to the *cis*-side of the Golgi apparatus, processes mediated by tethering factors and complexes of ERGIC- and Golgi-associated SNARE proteins (Brandizzi and Barlowe, 2013).

Cargo subsequently moves through the Golgi complex, where it can undergo post-translational modification and processing, most notably at the level of glycosylation, by enzymes that each localise within a narrow range of Golgi cisternae. How cargo moves forward is controversial, but the current consensus is that a Golgi cisterna moves ‘en bloc’, with cargo encountering Golgi-resident enzymes, as these are distilled backwards via selective incorporation into COPI vesicles (Pantazopoulou and Glick, 2019). COPI works analogously to COPII, with vesicle production initiated by the activation and membrane anchoring of ADP-ribosylation factor (ARF) 1 GTPase (Beck et al., 2009). The COPI coat is recruited en masse, and includes moieties that bind cargo and cargo adaptors, and those that scaffold the assembly and induce membrane curvature. Meanwhile, ARF GTPase activating proteins (ARF-GAPs) sense completion of COPI budding, and facilitate coat disassembly. Conserved oligomeric Golgi complex (COG) is a crucial membrane-tethering complex for COPI vesicles, working in conjunction with RAB GTPases and golgin coiled-coil proteins, while membrane fusion involves Golgi-specific SNAREs (Fisher and Ungar, 2016). In addition to intra-Golgi transport, COPI also recycles proteins from the ERGIC and Golgi apparatus back to the ER (Brandizzi and Barlowe, 2013).

Proteins exit the Golgi at the *trans*-Golgi network en route to the cell surface or towards the endosomal system (discussed below) (De Matteis and Luini, 2008). In the case of secretory/surface cargo, where export is constitutive, carriers appear to be tubular. In contrast, cargoes subject to regulated secretion are concentrated into specialised granules, which fuse with the surface in a Ca^2+^-regulated manner (Anantharam and Kreutzberger, 2019). SNARE-mediated fusion of these granules with the cell surface is facilitated by a range of specialised accessory proteins, including members of the synaptotagmin family of Ca^2+^ sensors.

## The endocytic pathway

Surface membrane proteins define the interface between cells and their environment, and cells constantly refine the population of proteins at the surface via rounds of endocytosis and subsequent endosomal sorting (see poster). Endocytosis also brings in soluble proteins, either as ligands to surface receptors or as bulk-flow constituents. The best-characterised uptake pathway is clathrin-mediated endocytosis (McMahon and Boucrot, 2011). Here, clathrin provides the membrane-deforming scaffold, the assembly of which onto the plasma membrane is mediated by cargo-binding adaptor complexes. The best known of these is adaptor protein complex (AP) 2, which is part of a wider family of hetero-tetrameric adaptor complexes, AP1-5. AP2 binds to peptide motifs within the cytoplasmic domains of a range of membrane proteins, while also binding clathrin. Other clathrin adaptors engage client cargoes more selectively. Meanwhile, numerous accessory proteins promote key steps towards vesicle formation, leading ultimately to the recruitment of the scission GTPase, dynamin. Clathrin-coated vesicle formation also relies on local actin dynamics, and on the local generation of phosphatidylinositol 4,5-bisphosphate [PtdIns(4,5)P_2_] which aids both actin and coat protein recruitment. PtdIns(4,5)P_2_ phosphatases, notably synaptojanin, complete the vesicle cycle.

Other endocytic mechanisms employ membrane-deforming proteins that selectively engage client membrane cargo while often utilising actin to provide a driving force (Sandvig et al., 2018). Examples include those mediated by flotilin, endophilin and cell division control protein 42 homologue (CDC42). Caveolae, comprised of the membrane protein caveolin and the structural protein cavin, provide a prominent and clinically important example of plasma membrane invagination (Parton, 2018). They primarily appear to function as a reservoir for surface membrane that forms or is dissipated according to alterations in membrane tension, and they are particularly enriched in elastic tissues such as the lung and muscle. Their role may also extend to the sequestration of some signalling pathway components. Caveolae can also undergo endocytosis, although the mechanisms remain poorly defined (Parton, 2018).

Endocytic vesicles fuse to form early endosomes, which are the major sorting stations within the endocytic pathway. The early (or sorting) endosomes are defined by the presence of RAB5 and phosphatidylinositol 3-phosphate (PtdIns3P), which promote the recruitment of numerous effector proteins to the endosomal membrane (Wandinger-Ness and Zerial, 2014). Eventually, endosomes mature as RAB5 is replaced with RAB7 and PtdIns3P is converted to phosphatidylinositol 3,5-bisphosphate [PtdIns(3,5)P_2_] to generate late endosomes. These fuse with and discharge into lysosomes, leading to the digestion of lumenal content (Wartosch et al., 2015)

To allow the degradation of integral membrane proteins, these must move from the endosomal limiting membrane into the lysosomal lumenal space. Hence, these membrane proteins are incorporated into intralumenal vesicles (ILVs), giving rise to the multivesicular body (MVB). The signal for ILV sorting, K63-linked polyubiquitin, is recognised by a series of endosomal sorting complexes required for transport (ESCRT) complexes and accessory factors (Christ et al., 2017), of which ESCRT-0 and ESCRT-I form the principal ubiquitin receptors. Cargo is passed onwards to ESCRT-III, a membrane-deforming polymer that combines with the AAA ATPase vacuolar protein sorting (VPS) 4 to mediate membrane fission and ILV completion.

Endocytic cargo can escape from the MVB-lysosome pathway by recycling to the cell surface or diverting to the Golgi complex (Cullen and Steinberg, 2018). These pathways involve the formation of tubular or vesicular intermediates that bud away from the endosome. The retriever and retromer complexes are important players in recycling from the sorting endosome that interact with sorting nexin proteins. Recycling to the plasma membrane can occur via a ‘fast’ direct route, or a ‘slow’ indirect route by which cargo is first delivered to the recycling endosome, marked by RAB11, and utilises a distinct set of molecular machineries such as EH domain-containing protein 1 (EHD1) and molecule interacting with CasL protein-like 1 (MICAL-L1), which remain less well characterised than those at the sorting endosome (Goldenring, 2015).

Synaptic vesicles are the mediators of neurotransmitter release at neuronal synapses. Synaptic vesicle biogenesis within the nerve terminal can occur directly from the plasma membrane via endocytosis, or from pre-existing endosomes through selective budding from this compartment (Saheki and De Camilli, 2012), and is therefore highly dependent upon the endocytic trafficking machinery. Fusion of synaptic vesicles with the plasma membrane for neurotransmitter release is tightly regulated, and occurs in a similar way to the regulated exocytosis of secretory granules described above, being mediated by SNAREs and controlled by Ca^2+^ sensors (Südhof, 2013).

## Diseases that are caused by defective membrane traffic

Diseases associated with defective membrane traffic collectively manifest in practically all tissues and organ systems, with some affecting multiple systems and others restricted to one tissue type or organ. Diseases most often arise from mutations that cause loss of expression or function of transport machinery components, but some are caused by toxic gain-of-function mutations. Diseases attributable to defective trafficking machinery can be developmental in nature, or can arise during the lifespan, often manifesting during ageing. Here, we categorise membrane trafficking-related diseases based upon their tissue and organ system involvement (also see poster). The discussion is not exhaustive; for a more comprehensive list of diseases associated with defective trafficking please consult Table 1.

**Table 1.**
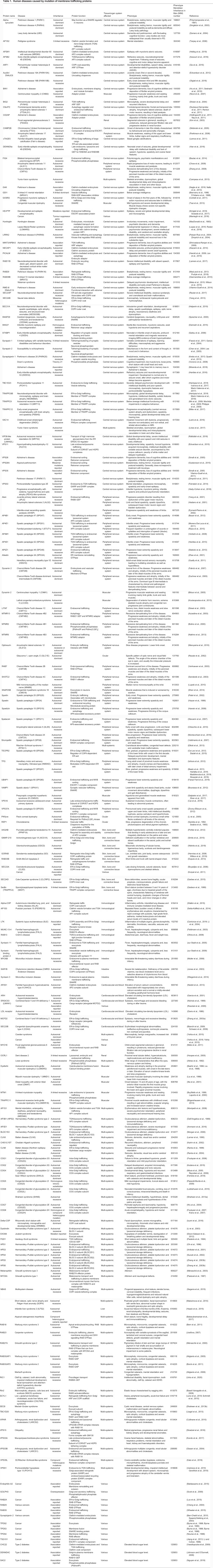
**Human diseases caused by mutation of membrane trafficking proteins**

### Neurological disease

Major neurodegenerative diseases are strongly associated with defects in membrane traffic, particularly within the endosomal system (Schreij et al., 2016). Genetic association studies link variants or altered expression levels of the clathrin-mediated endocytosis components phosphatidylinositol-binding clathrin assembly protein (PICALM) (Harold et al., 2009; Jun et al., 2010), bridging integrator 1 (BIN1)/amphiphysin 2 (Hu et al., 2011; Seshadri et al., 2010), cortactin-CD2-associated protein (CD2AP) (Hollingworth et al., 2011; Naj et al., 2011) and synaptojanin (McMahon and Boucrot, 2011; Miranda et al., 2018), with the risk of acquiring Alzheimer's disease (AD). Additionally, deficiency in VPS26 and VPS35, two subunits of the retromer complex for endosomal recycling, has been observed in AD (Small et al., 2005). In AD, differences in endocytic trafficking and processing of amyloid precursor protein (APP) to its cytotoxic product Aβ can explain the involvement of endocytic traffic in AD pathogenesis (Toh and Gleeson, 2016). Endocytic traffic may also affect AD pathogenesis in other ways; for example, by influencing the susceptibility of neurons to Aβ (which itself can disrupt endocytic traffic), the uptake of toxic Aβ aggregates from the cell exterior, the production of synaptic vesicles or abundance of post-synaptic receptors, or by altering lysosome homeostasis and autophagy pathways that are important for cell viability (Nixon, 2017). Increased processing of APP to Aβ may also arise from altered trafficking at the Golgi apparatus, although the molecular details are less clear (Joshi and Wang, 2015).

Parkinson's disease (PD) is also strongly associated with defective endocytic traffic, including the mutation or altered expression of various endocytic components (Abeliovich and Gitler, 2016). These include cyclin G-associated kinase (GAK) (Nagle et al., 2016; Nalls et al., 2014), auxilin (Edvardson et al., 2012; Olgiati et al., 2016) and synaptojanin (Krebs et al., 2013; Quadri et al., 2013) [which function in clathrin-mediated endocytosis (McMahon and Boucrot, 2011)], the retromer subunit VPS35 (Vilarino-Guell et al., 2011; Zimprich et al., 2011) and the retromer-associated protein receptor-mediated endocytosis 8 (RME-8) (Vilarino-Guell et al., 2014). As in AD, endocytic traffic may lead to PD pathology in several ways; for example, by influencing the uptake of toxic α-synuclein aggregates, by altering synaptic vesicle or neurotransmitter receptor traffic, or by affecting lysosome homeostasis and autophagy (Abeliovich and Gitler, 2016). Of note, mutations of several lysosomal proteins are strongly associated with PD (Dehay et al., 2013), as is defective autophagic clearance of mitochondria (Ryan et al., 2015).

Defective traffic in the early secretory pathway is also relevant for PD pathogenesis. RAB39B, a mutation of which is associated with early-onset PD as well as X-linked intellectual disability (Giannandrea et al., 2010; Mata et al., 2015; Wilson et al., 2014), is required for ER-to-Golgi transport of the synaptic α-amino-3-hydroxy-5-methyl-4-isoxazolepropionic (AMPA) receptor subunit GluA2 (Mignogna et al., 2015). Interestingly, excess α-synuclein can also disrupt ER-to-Golgi traffic, most likely at the level of COPII vesicle tethering or fusion (Cooper et al., 2006; Thayanidhi et al., 2010). α-Synuclein appears to normally function in synaptic vesicle fusion (Burre et al., 2010; Chandra et al., 2005), hence its aggregation or loss of function likely also directly affect neurotransmitter release (Carstea et al., 1997; Polymeropoulos et al., 1997; Singleton et al., 2003). As for AD, PD pathology may arise from defects in other trafficking pathways. A protein of much current interest is leucine-rich repeat kinase 2 (LRRK2), which is mutated in ∼1% of sporadic and ∼5% of familial PD (Paisan-Ruiz et al., 2004; Zimprich et al., 2004). LRRK2 phosphorylates several RAB GTPases (Steger et al., 2016), functioning in diverse trafficking steps, and the most common PD mutations cause LRRK2 activation (West et al., 2005). Excessive LRRK2-mediated phosphorylation alters the ability of these RABs to engage with regulatory factors and effector proteins, thereby disrupting traffic (Steger et al., 2016). Of interest, RAB29 stimulates LRRK2 activation at cellular membranes (Gomez et al., 2019; Purlyte et al., 2018), and is also independently linked to PD, indicating that these proteins (co)operate in a common disease pathway (MacLeod et al., 2013).

Frontotemporal dementia (FTD) is a neurodegenerative disease that is commonly associated with early onset of symptoms (Warren et al., 2013). Mutation of C9orf72, which encodes a RAB GEF, is strongly associated with familial FTD (DeJesus-Hernandez et al., 2011; Renton et al., 2011). Expansion of nucleotide repeats may cause a toxic gain of function at the RNA level, whereas a loss of protein function may also contribute to FTD pathology by altering trafficking to the lysosome, with likely downstream effects upon autophagy (Balendra and Isaacs, 2018). Consistent with this, FTD can be caused by mutation of the ESCRT-III subunit charged multivesicular body protein 2B (CHMP2B), which is involved in MVB sorting (Skibinski et al., 2005).

Amyotrophic lateral sclerosis (ALS), or motor neuron disease, results in progressive degeneration of motor neurons (Hardiman et al., 2017). ALS and FTD represent two extremes of a phenotypic spectrum, and share common pathogenic mechanisms (Ferrari et al., 2011). Thus, C9orf72 mutation causes ALS as well as FTD (DeJesus-Hernandez et al., 2011; Renton et al., 2011). Other endocytic proteins are mutated in ALS, including the RAB5 GEF Alsin (also known as ALS2) and the inositol phosphatase factor-induced gene 4 (FIG4) (Chow et al., 2009; Yang et al., 2001). Dysregulation of endocytic transport, in turn affecting lysosome function and autophagy, is therefore associated with ALS. Of note, mutation of several ALS-associated proteins, including superoxide dismutase 1 (SOD1), RNA-binding protein fused in sarcoma (FUS) and TAR DNA-binding protein 43 (TDP43; also known as TARDBP), have been reported to disrupt the secretory pathway, suggesting additional mechanisms linking defective traffic to ALS (Soo et al., 2015).

Charcot Marie-Tooth (CMT) disease is a genetically and clinically diverse group of peripheral neuropathies (Rossor et al., 2013). Most CMT forms result from altered expression or mutation of myelin components, but mutation of several endocytic proteins is also a cause. For example, recessive demyelinating forms of CMT (CMT4) result from mutation of the endocytic recycling protein SH3 domain and tetratricopeptide repeats 2 (SH3TC2) (Senderek et al., 2003), as well as the myotubularins and FIG4, which influence traffic by acting upon endosomal phosphoinositides (Azzedine et al., 2003; Bolino et al., 2000; Nakhro et al., 2013; Zhang et al., 2008). Lipopolysaccharide-induced tumour necrosis factor alpha factor (LITAF), a protein involved in endocytic protein sorting, causes the autosomal dominant demyelinating CMT1 (also known as SIMPLE) (Street et al., 2003). Meanwhile, mutation of RAB7 causes a dominant axonal form of CMT (Verhoeven et al., 2003).

Similarly, mutation of endocytic factors is associated with hereditary spastic paraplegia (HSP), a genetically diverse disorder that manifests as progressive loss of lower limb movement control (Blackstone et al., 2011). Notable HSP-involved proteins are spastin (SPG4; also known as SPAST), which couples membrane remodelling with microtubule dynamics, including during endocytic traffic (Hazan et al., 1999), spartin (SPG20; also known as SPART), an endosomal protein that also associates with microtubules (Patel et al., 2002), and strumpellin (SPG8; also known as WASHC5), which is part of the WASH WASP and SCAR (WASH) homologue complex that operates in retromer-mediated endocytic recycling (Valdmanis et al., 2007). Interestingly, spastin also functions at the ER, and mutations in Atlastin (also known as ATL1), another ER membrane remodelling protein, also cause HSP (Zhao et al., 2001). It is currently unclear how changes in ER morphology lead to HSP. Another group of HSPs is caused by mutations within subunits of the AP4 and AP5 adaptor complexes that function in post-Golgi trafficking (Bauer et al., 2012; Hardies et al., 2015; Moreno-De-Luca et al., 2011; Slabicki et al., 2010; Verkerk et al., 2009), which likely affect endolysosomal function (Sanger et al., 2019). Interestingly, AP4 is important for trafficking of the autophagy-initiating factor ATG9A, suggesting a link between HSP and dysregulated autophagy (Mattera et al., 2017; Davies et al., 2018). HSP also results from mutations in ubiquitin-associated protein 1 (UBAP1) and VPS37A (Farazi Fard et al., 2019; Zivony-Elboum et al., 2012), components of ESCRT-I required for MVB sorting (Schmidt and Teis, 2012). Mutations in Trk-fused gene (TFG) and tectonin beta-propeller repeat-containing protein 2 (TECPR2), proteins that associate with COPII and help mediate ER-to-Golgi transport, also cause HSP, indicating that defective trafficking in the early secretory pathway can also cause this type of disorder (Beetz et al., 2013; Stadel et al., 2015).

Mutations in the endocytic machinery are prevalent in other rare neurological disorders (Table 1). The consequent defects in endocytosis and endosomal recycling may alter presynaptic vesicle biogenesis or postsynaptic neurotransmitter receptor availability. Meanwhile, defects in the later stages of the endocytic pathway can affect lysosome homeostasis and autophagy, which, if impaired, result in cytotoxic stress. Defective traffic in the secretory pathway is also associated with several neurological diseases. Here, defective transport may alter axon and dendrite morphogenesis, affect the surface levels of neurotransmitter receptors, or induce cytotoxic ER stress due to cargo accumulation in this compartment.

### Ocular disease

Eye pathology has been reported in several trafficking-related multi-systemic disorders, including the ciliopathies and the X-linked Lowe syndrome, which are described below. Choroideremia, which is an eye-specific disorder, manifests as degeneration of rod photoreceptors and retinal pigment epithelial cells (Moosajee et al., 2014). It is caused by mutations in RAB escort protein 1 (REP-1), a chaperone required for the prenylation of all RABs, grossly disrupting membrane traffic in the affected cells (Alory and Balch, 2001; Sankila et al., 1992; Seabra et al., 1993). The retinal tissue-restricted nature of choroideremia is likely because a second RAB escort protein, REP-2, compensates for the loss of REP-1 in other cell types, but is not expressed in the retina (Cremers et al., 1994).

### Skin, bone and connective tissue disorders

The extracellular matrix, which surrounds cells in our skin, bone and connective tissues, is a major secreted product in the human body. Consequently, matrix-rich tissues appear particularly susceptible to mutations affecting the secretory pathway that disrupt matrix deposition. Mutations in SEC23A, a component of the COPII coat, cause the skeletal disorder cranio-lentico-sutural dysplasia (CLSD) (Boyadjiev et al., 2006). Although COPII is essential for secretion, CLSD is tissue restricted, because most cells also express the functionally analogous SEC23B, sustaining COPII functionality (Khoriaty et al., 2018). Mutations in Sedlin (also known as TRAPPC2), a component of the transport protein particle (TRAPP) complex operating between the ER and Golgi, a RAB GEF and possible vesicle-tethering factor (Barrowman et al., 2010), cause X-linked spondyloepiphyseal dysplasia tarda (SEDT) (Gedeon et al., 1999). Sedlin also regulates SAR1, and both CLSD and SEDT mutations give rise to defective procollagen export from the ER, causing matrix defects and skeletal dysplasia (Boyadjiev et al., 2011; Venditti et al., 2012). Null and hypomorphic mutations in the Golgi vesicle-tethering factor Golgi microtubule-associated protein of 210 kDa [GMAP-210; also known as thyroid hormone receptor interactor 11 (TRIP11)] are responsible for the lethal skeletal dysplasia achondrogenesis type 1A (ACG1A) and the milder odontochondrodysplasia (ODCD), respectively (Smits et al., 2010; Wehrle et al., 2019). In both cases, the major pathogenic mechanism is defective traffic and improper glycosylation of matrix proteins within the Golgi (Smits et al., 2010; Wehrle et al., 2019). GMAP-210 is also important for cargo traffic to the primary cilium (Follit et al., 2008), and the phenotype may therefore partly arise from defective ciliary signalling that is required to maintain chondrocyte differentiation (Wang et al., 2013). Similarly, mutations in the *trans*-Golgi protein RAB6-interacting golgin (GORAB), which functions in COPI-mediated traffic, cause the skin and bone disorder gerodermia osteodysplastica, likely as a consequence of disrupted matrix protein glycosylation (Hennies et al., 2008; Witkos et al., 2019). This not only affects matrix assembly, but is also important for controlling TGFβ (also known as TGFB1) signalling to prevent cell senescence (Chan et al., 2018). Mutations in Golgi RAB33B cause Smith-McCort syndrome (Dupuis et al., 2013), an osteochondrodysplasia. This is likely due to defects in Golgi traffic and autophagosome formation, both RAB33B-dependent processes (Morgan et al., 2019).

### Immunological disease

Membrane traffic is vital for innate and adaptive immunity; for example, in mediating phagocytosis of invading microorganisms, supporting the biosynthesis and signalling of the many receptors found on immune cells, and facilitating the secretion of antibodies, cytokines and other immunomodulatory factors. Consequently, several immunological diseases, including immunodeficiencies and autoimmune disorders, can be attributed to defective membrane trafficking. These include familial haemophagocytic lymphohistiocytosis, an immune disorder caused by mutations in protein unc-13 homologue D (MUNC-13-4; also known as UNC13D) (Feldmann et al., 2003), syntaxin 11 (zur Stadt et al., 2005) or syntaxin binding protein 2 (zur Stadt et al., 2009). These proteins control lytic granule release at the T-cell and natural killer (NK) cell immune synapse and platelet granule exocytosis. As a result, cells with these mutations have a compromised ability to mediate cell killing, leading to hyperactivation of the immune system (Gholam et al., 2011). Another interesting example is leukocyte tyrosine kinase receptor (LTK), an ER-associated tyrosine kinase that controls COPII assembly and ER-to-Golgi traffic (Centonze et al., 2019). Gain-of-function mutations in LTK are associated with the autoimmune disorder systemic lupus erythematosus, and it has been proposed that increased LTK activity, and therefore increased COPII-mediated ER export, allows plasma cells to cope better with the increased production and secretion of autoantibodies, thereby contributing to the autoimmune phenotype seen in lupus (Centonze et al., 2019; Li et al., 2004).

### Intestinal disorders

Defective traffic within both the secretory and endocytic pathways can affect enterocyte function and cause intestinal disease. Enterocytes absorb fats from the intestine and package them into chylomicron particles, which form at the ER and are secreted into the bloodstream. Chylomicron retention disease is a rare disorder caused by mutation in SAR1B (Jones et al., 2003), which impairs chylomicron particle export from the ER, reducing their secretion and the availability of fats and fat-soluble vitamins throughout the body (Roy et al., 1987). The disease is restricted to enterocytes, most likely because the paralogue SAR1A fulfils SAR1 function in other cell types. Microvillus inclusion disease also affects enterocytes, with a loss of microvilli from the apical surface and impaired nutrient absorption (Davidson et al., 1978). It is caused by mutations in the actin motor myosin (MYO) 5B, which is required for endosomal recycling to the apical membrane (Muller et al., 2008), or in syntaxin 3, which is required for vesicle fusion at the apical membrane (Wiegerinck et al., 2014).

### Liver disease

Membrane trafficking is important in hepatocytes, which secrete a multitude of proteins into the bloodstream. Mutation of SCY1-like pseudokinase 1 (SCYL1) or neuroblastoma-amplified sequence (NBAS), which function in COPI vesicle traffic, can manifest in the liver, but typically also affect other tissues, and are discussed further in the ‘Multi-systemic disorders’ section below.

### Cardiovascular disease and blood disorders

Cholesterol is transported in the blood as low-density lipoprotein (LDL) particles. These are internalised, particularly into hepatocytes, by receptor-mediated endocytosis. Defective LDL uptake causes hypercholesterolaemia, which can manifest as atherosclerosis and premature coronary heart disease (Brown and Goldstein, 1986). Mutations in the LDL receptor (LDLR) that abolish LDL binding have been reported, but of more relevance to this article are LDLR mutations that disrupt binding to disabled homologue 2 (DAB2) and autosomal recessive hypercholesterolaemia (ARH; also known as LDLRAP1), adaptor proteins that mediate LDLR uptake by clathrin-dependent endocytosis (Davis et al., 1986; He et al., 2002; Maurer and Cooper, 2006). Similarly, mutation of ARH itself can also cause hypercholesterolaemia (Garcia et al., 2001).

Defects within the secretory pathway can affect red blood cell production and the production of clotting factors. In the former, mutations within the COPII subunit SEC23B cause congenital dyserythropoietic anaemia type II (Bianchi et al., 2009; Schwarz et al., 2009), likely due to perturbation of ER-to-Golgi traffic in erythroblasts that impairs the delivery and glycosylation of proteins required for red blood cell formation (Denecke and Marquardt, 2009). The widespread expression of the paralogue SEC23A likely accounts for the restricted phenotype of SEC23B mutation. The blood clotting disorder combined factor V and VIII deficiency results from mutations in multiple coagulation factor deficiency 2 (MCFD2) and lectin mannose binding 1 (LMAN1) (Nichols et al., 1998; Zhang et al., 2003a). MCFD2 and LMAN1 combine to form a cargo receptor for the ER-to-Golgi transport of blood clotting factors V and VIII and thus are essential for their secretion (Zhang et al., 2005).

### Renal disorders

Renal dysfunction occurs in several multi-systemic trafficking disorders, most notably the ciliopathies. Mutations in the actin-associated proteins CD2AP and MYO1E cause focal segmental glomerulosclerosis, which progressively reduces the ability of the glomerulus to filter the blood, ending in renal failure (Kim et al., 2003; Mele et al., 2011). Both proteins participate in endocytosis, which is required to maintain podocyte foot processes and thus effective filtration, but whether defective traffic constitutes a disease mechanism is unclear (Inoue and Ishibe, 2015). The proteins may directly act upon actin within the foot processes (Inoue and Ishibe, 2015), while CD2AP is also a component of the slit diaphragm (Shih et al., 2001). Dent disease and cystinosis are proximal tubulopathies in which the ability of the proximal tubule to re-absorb proteins by endocytosis is disrupted (Ivanova et al., 2015; Piwon et al., 2000; Wang et al., 2000). However, in both diseases, the mutations are not in the trafficking machinery; Dent disease is caused by mutation in the endosomal chloride channel chloride channel protein 5 (ClC-5; also known as CLCN5) (Lloyd et al., 1996), and cystinosis by mutation of a lysosomal cystine transporter (Town et al., 1998).

### Muscular disorders

Centronuclear myopathies (CNMs) are a group of muscle disorders that derive their name from centrally located muscle cell nuclei. Mutations in the membrane fission protein dynamin 2 or in BIN1, a BAR domain protein able to sculpt membrane shape, cause congenital CNM (Bitoun et al., 2005; Nicot et al., 2007). These two proteins, which physically interact, participate in endocytosis in most cells (Takei et al., 1999). However, in muscle, they are critical for the formation and maintenance of T-tubules, membrane invaginations that penetrate into muscle cells (Chin et al., 2015; Lee et al., 2002). Thus, disruption of T-tubule morphogenesis and function is a major disease mechanism in CNMs. Mutation of myotubularin 1 (MTM1), a member of the myotubularin family of endosomal inositol phosphatases, causes an X-linked CNM (Buj-Bello et al., 1999; Laporte et al., 2000). MTM1 binds to BIN1 (Royer et al., 2013), and, as seen in congenital CNMs, MTM1 mutation disrupts T-tubules, indicating a likely common disease mechanism (Al-Qusairi et al., 2009; Dowling et al., 2009). Mutation of dysferlin, a Ca^2+^-binding protein with homology to the membrane fusion regulator synaptotagmin, causes muscular dystrophy (Bashir et al., 1998; Illa et al., 2001; Liu et al., 1998). The likely pathological mechanism is disruption of muscle cell integrity due to a defect in vesicle fusion and repair of the plasma membrane (Bansal et al., 2003; Lek et al., 2012). Mutations in two proteins required for ER-to-Golgi transport, the TRAPP complex subunit TRAPPC11, and the SNARE Golgi SNAP receptor complex member 2 (GOSR2), are also linked to muscular dystrophy (Bögershausen et al., 2013; Tsai et al., 2013). Hypoglycosylation of α-dystroglycan occurs in both cases (Larson et al., 2018). Because α-dystroglycan glycosylation is important for linking the muscle sarcolemma to the extracellular matrix (Barresi and Campbell, 2006), these glycosylation defects can explain the destabilisation of muscle fibres seen in patients.

### Multi-systemic disorders

There are numerous multi-systemic disorders associated with mutations in the membrane trafficking machinery (Table 1). Several belong to larger disease classes such as congenital disorders of glycosylation (CDGs), ciliopathies and lysosomal storage disorders (LSDs). Many CDGs are caused by loss of Golgi glycosylation enzyme or ion or sugar transporter activity, but mutations within the COG vesicle-tethering complex account for several (Ng and Freeze, 2018). Here, impaired COPI-dependent recycling of glycosylation enzymes in the Golgi stack leads to their inefficient retention, affecting the glycosylation of proteins and lipids (Fisher and Ungar, 2016).

The ciliopathies are a large disease class associated with loss of cilia or defective ciliary signalling (Reiter and Leroux, 2017). The commonly affected tissues include the brain, retina and kidney. Several ciliopathies are associated with defective transport of proteins to or within the cilium, although the latter is not vesicle mediated (Reiter and Leroux, 2017). Vesicle-mediated transport from the Golgi apparatus to the cilium is important for the generation and maintenance of cilia, with RAB8 and its effector, the exocyst vesicle-tethering complex, constituting the key machinery of this trafficking step (Hsiao et al., 2012). Indeed, mutations in two exocyst subunits, exocyst complex component 84 (EXO84; also known as EXOC8) and SEC8 (also known as EXOC4), have been found in the ciliopathies Joubert syndrome and Meckel–Gruber syndrome (Dixon-Salazar et al., 2012; Shaheen et al., 2013a,b). Intraflagellar transport protein 20 (IFT20) is an important player in Golgi-to-cilium transport of certain membrane proteins (Follit et al., 2008; Monis et al., 2017), and mutation of VPS15, which also causes a ciliopathy, impairs this transport pathway (Stoetzel et al., 2016). Interestingly, IFT20 is anchored to the Golgi by GMAP-210 (Follit et al., 2008), suggesting that the two skeletal dysplasias caused by GMAP-210 mutation (ACG1A and ODCD, discussed above) may have a ciliary component (Smits et al., 2010; Wehrle et al., 2019).

LSDs are a third broad class of disease, defined by impaired lysosome-mediated degradation (Platt et al., 2018). Many LSDs result from the loss of hydrolase expression, but some involve defective hydrolase trafficking. For example, ceroid-lipofuscinosis, neuronal 8 (CLN8) is a cargo receptor for trafficking of newly synthesised hydrolases from the ER to the Golgi (di Ronza et al., 2018), and mutations in CLN8 cause the LSD Batten disease (Ranta et al., 1999). Mutation of VPS33A, a common component of the class C core vacuole/endosome tethering (CORVET) and homotypic fusion and protein sorting (HOPS) multi-subunit vesicle-tethering complexes that operate at the early and late endosome/lysosome, respectively, causes the LSD mucopolysaccharidosis (Kondo et al., 2017).

Lysosome-related organelles (LROs) are found in specific cell types and carry out specialised functions (Marks et al., 2013). Examples include melanosomes in skin melanocytes and retinal pigment epithelial cells, which are important for pigmentation, lytic granules of NK and T-cells that mediate target cell killing, and Weibel–Palade bodies in endothelial cells that contribute to blood clotting. Chediak–Higashi, Griscelli and Hermansky–Pudlak syndromes are all associated with defective LRO biogenesis, and in many cases are due to defects in the relevant LRO trafficking machinery (Huizing et al., 2008). For example, Griscelli syndrome, characterised by hypopigmentation and immunodeficiency, can be caused by mutations in RAB27A, its effector melanophilin, or the actin motor MYO5A, which together facilitate melanosome movement to the cell periphery for delivery of pigment to neighbouring keratinocytes (Ménasché et al., 2003; Ménasché et al., 2000; Pastural et al., 1997). Hermansky–Pudlak syndrome, which presents as hypopigmentation, bleeding and additional symptoms depending on the subtype, is caused by mutations in subunits of the biogenesis of lysosome-related organelle complex (BLOC)-1 (Li et al., 2003; Morgan et al., 2006), BLOC-2 (Anikster et al., 2001; Zhang et al., 2003b), BLOC-3 (Oh et al., 1996; Suzuki et al., 2002) or AP3 (Ammann et al., 2016; Dell'Angelica et al., 1999) complexes that are involved in transport of cargo proteins from endosomes to LROs. Chediak–Higashi syndrome, which manifests as albinism, excessive bleeding and immunodeficiency, is caused by mutations in lysosomal trafficking regulator (LYST) (Karim et al., 2002), which appears to function in endolysosomal trafficking (Gil-Krzewska et al., 2016).

Lysosome dysfunction has also been reported in the rare X-linked disorder Lowe syndrome, which affects the brain, eyes and kidneys, and is caused by mutation of the inositol phosphatase occulocerebrorenal Lowe syndrome protein (OCRL) (Attree et al., 1992). The aetiology of Lowe is complex, since build-up of the OCRL substrate PtdIns(4,5)P_2_ disrupts not only lysosomal function, which results in an additional autophagy defect, but also affects endocytosis, endocytic recycling and trafficking to the cilium (De Matteis et al., 2017). Hence, disruption of several trafficking steps is likely to cause the Lowe syndrome phenotypes seen in patients. Interestingly, mutations in OCRL also cause Dent-2 disease, for which the symptoms are largely restricted to the kidney (Hoopes et al., 2005). The reasons for this dual pathophenotype remain unclear.

Defective COPI-dependent recycling from the Golgi apparatus to the ER is associated with two multi-systemic genetic disorders, both affecting the liver. Mutation of the COPI accessory protein SCYL1 causes low γ-glutamyl-transferase cholestasis, acute liver failure, and neurodegeneration (CALFAN) syndrome, manifesting as hepatocyte death and liver failure, as well as ataxia resulting from cerebellar neurodegeneration (Lenz et al., 2018; Schmidt et al., 2015). Mutation of NBAS, a component of the NBAS/RINT1/ZW10 (NRZ) ER-localised COPI vesicle-tethering complex results in a nearly identical liver phenotype, and also causes bone, connective tissue, retina and immune system defects (Balasubramanian et al., 2017; Haack et al., 2015; Maksimova et al., 2010; Segarra et al., 2015). These findings suggest a high requirement for the secretory pathway in the affected cell types, including hepatocytes, consistent with them secreting large amounts of material into the bloodstream.

### Cancer

Membrane trafficking is intimately linked with cancer, with trafficking in both the secretory and endocytic pathways playing an important role in many types of cancer. Endocytic trafficking is responsible for the abundance and signalling capacity of mitogenic receptors, adhesion molecules and immune modulators that determine the ability of the immune system to detect cancer cells (Mellman and Yarden, 2013). Hence, changes in the expression levels or degree of phosphorylation of endocytic trafficking machinery can correlate with cancer susceptibility or prognosis. In addition, cancer-causing mutations within the components of the endocytic machinery have been described. A recent example is RAB35, which mediates various endocytic trafficking steps (Klinkert and Echard, 2016). Oncogenic mutations in RAB35, although extremely rare, have been shown to cause its constitutive activation and promiscuous growth factor signalling from endosomal compartments (Wheeler et al., 2015). Altered expression and splicing of tumour susceptibility gene 101 (TSG101), has been found in cancer (Jiang et al., 2013), whereby impaired growth factor receptor downregulation at the endosome may contribute to tumourigenesis (Lu et al., 2003). Interestingly, toxic gain-of-function mutation of p53 can also promote the recycling of integrins and growth factor receptors, which is responsible for increased cell migration and metastatic potential of tumour cells (Muller et al., 2009).

The secretory pathway can influence cancer susceptibility and disease progression in a number of ways (Dejeans et al., 2014). We know that cell surface glycans, which are generated within the secretory pathway, are important for processes contributing to cancer development and metastasis, including signalling, adhesion and migration (Pinho and Reis, 2015). A particularly interesting example of an oncogenic trafficking protein is Golgi phosphoprotein 3 (GOLPH3), which is highly expressed in several cancers (Scott et al., 2009). GOLPH3 appears to participate in intra-Golgi transport, which is required for Golgi enzyme retention and correct protein glycosylation (Ali et al., 2012; Chang et al., 2013; Isaji et al., 2014; Pereira et al., 2014), as well as export of cargo from the *trans*-Golgi (Rahajeng et al., 2019). GOLPH3 overexpression stimulates a number of mitogenic signalling pathways, which may be a consequence of altering the cell surface glycan profile and thus the signalling capacity of surface receptors (Rizzo et al., 2017). In addition, GOLPH3 has been implicated in a DNA stress response pathway, linking DNA damage to the Golgi apparatus (Farber-Katz et al., 2014). In this context, GOLPH3 overexpression can promote cell survival upon DNA damage, which may be relevant to the cancer phenotype. Another interesting example is mutation of the ER-to-Golgi trafficking protein LMAN1 in colorectal cancers, which causes reduced secretion of the LMAN1 client protein α-1-antitrypsin (A1AT; also known as SERPINA1), an angiogenesis inhibitor, thereby contributing to tumour blood supply and growth (Roeckel et al., 2009).

### Diabetes

Exocytosis of insulin from pancreatic beta cells, and the endocytic and secretory trafficking of insulin receptors and glucose transporters in target cells, may all directly affect diabetes susceptibility or progression. For example, the inositol phosphatase suppressor of actin 2 (SAC2; also known as INPP5F) functions in insulin granule exocytosis from pancreatic beta cells, and its levels are reduced in type II diabetic patients, suggesting that SAC2 insufficiency might contribute to impaired insulin release in these patients (Nguyen et al., 2019). Another protein of interest is clathrin heavy chain 22 (CHC22), which is involved in the trafficking of glucose transporter type 4 (GLUT4; also known as SLC2A4) in muscle and fat cells, where it mediates glucose uptake in response to insulin signalling (Vassilopoulos et al., 2009). Two CHC22 variants exist in the human population, which differ in their ability to traffic GLUT4 and thus remove glucose from the bloodstream (Fumagalli et al., 2019). The ‘new’ variant, which appeared later in evolution, increases cell surface levels of GLUT4 and glucose removal from the bloodstream, whereas the ‘older’ variant has a lower capacity to traffic GLUT4 to the cell surface and therefore to clear blood glucose. However, it remains to be seen whether people carrying the ‘older’ variant have a greater diabetes risk.

## Summary and conclusions

Membrane trafficking is a ubiquitous process and fundamentally important to all tissues. However, defects in components of the trafficking machinery often manifest as a tissue-specific phenotype. The nature of the observed defect depends upon the tissue expression of the trafficking component in question and its degree of functional redundancy, the rate-limiting trafficking steps within different cell types, and the abundance and types of cargo proteins expressed in different cells. The nature of the mutation itself is also important, as it can result in either a complete loss of expression or function of the trafficking component, a partial loss of expression or function, or, in some cases, a toxic overexpression or gain of function. This is expected to cause corresponding changes in the associated trafficking pathway(s), resulting in the observed phenotype. With regard to the tissue-specific nature of the diseases, it is interesting that the nervous system is particularly sensitive to disruption of the endolysosomal system, possibly due to the importance of endocytic traffic to maintain neurotransmission as well as the sensitivity of neurons to disrupted lysosome function and autophagy. Skin, bone and connective tissues are more sensitive to defective secretory traffic, reflecting the high secretory load in these tissues. Despite these generalisations, it is often hard to predict the phenotype one might expect upon mutation of a particular trafficking component, and understanding the disease mechanisms underlying most trafficking-related disorders is not trivial.

Defective traffic can manifest in a particular phenotype for several reasons. In some cases, it may be the failure to deliver a cargo protein to the correct destination compartment, causing dysfunction of that organelle, or the impaired ability of cells to secrete or internalise cargo effectively, resulting in systemic effects. In other cases, the inability to traffic proteins from their donor compartments may be problematic, as in the case of ER stress induction when proteins fail to exit this compartment. Similarly, the inability to degrade substrates by autophagy is cytotoxic. It is also worth noting that although impaired traffic can cause disease, in some contexts, trafficking might be required to sustain a disease phenotype. This appears to be true in cancer, where endocytic traffic is required to sustain proliferative signalling and cell migration, important for tumour growth and metastasis. Thus, in terms of developing therapeutics for trafficking disorders, a range of strategies is possible (Box 2). Gene therapy is one possible route, but drugs can potentially rescue defective organelle function. Therapeutic strategies could alleviate the cell stress that occurs downstream from organelle dysfunction, restore the disrupted trafficking step, or, in some cases, inhibit a transport step that is driving the disease phenotype. As we identify more rare diseases attributable to defects in membrane traffic, and better understand the mechanisms that underlie these and other more common disorders, we will undoubtedly be able to deliver better treatments and long-term therapies in the future.

**Box 2. Therapeutic approaches to rescue traffic-dependent phenotypes**

We lack effective therapies to treat most of the diseases associated with defective membrane trafficking. In principle, diseases caused by genetic mutation could be treated with gene therapy, but using this approach to successfully treat human disease remains in its infancy (Dunbar et al., 2018). A promising example is gene therapy for retinal dystrophy choroideremia, which is caused by loss of the RAB escort protein REP-1 (Sankila et al., 1992; Seabra et al., 1993). Here, *CHM*, the gene that encodes REP-1, is administered to the eye via a viral delivery vector. The therapy is currently undergoing phase 3 clinical trials following promising results in earlier stages of clinical testing (Xue et al., 2018). Many trafficking regulators, which are enzymes, are potentially amenable to treatment with small-molecule drugs. This approach remains to be explored more fully, but there is significant interest in targeting the protein kinase LRRK2 for treatment of Parkinson's disease (Zhao and Dzamko, 2019). Pathogenic LRRK2 mutations lead to overactive kinase activity and so chemically inhibiting this activity could protect against Parkinson's disease. As such, clinical trials are underway to test the safety and efficacy of LRRK2 inhibitors in human patients.
